# Major dietary patterns and sleep quality in relation to overweight/obesity among school children: A case-control study

**DOI:** 10.34172/hpp.2023.38

**Published:** 2023-12-16

**Authors:** Maedeh Massoudi, Bahram Pourghassem Gargari, Mohammad Asghari Jafarabadi, Solmaz Norouzi

**Affiliations:** ^1^Student Research Committee, Department of Biochemistry and Diet Therapy, Faculty of Nutrition and Food Sciences, Tabriz University of Medical Sciences, Tabriz, Iran; ^2^Nutrition Research Center, Department of Biochemistry and Diet Therapy, Faculty of Nutrition and Food Sciences, Tabriz University of Medical Sciences, Tabriz, Iran; ^3^Cabrini Research, Cabrini Health, Malvern, VIC, 3144, Australia; ^4^School of Public Health and Preventative Medicine, Faculty of Medicine, Nursing and Health Sciences, Monash University, Melbourne, VIC, 3004, Australia; ^5^Department of Psychiatry, School of Clinical Sciences, Faculty of Medicine, Nursing and Health Sciences, Monash University, Clayton, VIC, 3168, Australia; ^6^Road Traffic Injury Research Center, Tabriz University of Medical Sciences, Tabriz, Iran; ^7^Department of Biostatistics, Faculty of Medical Sciences, Tarbiat Modares University, Tehran, Iran; ^8^Department of Biostatistics and Epidemiology, Faculty of Medical Science, Zanjan

**Keywords:** Diet, Sleep quality, Sleep duration, Obesity, Overweight, Childhood

## Abstract

**Background:**

Childhood overweight/obesity is increasing worldwide. There is evidence on the role of dietary patterns (DPs) and sleep quality on body weight in adults, but studies on the association of major DPs, sleep quality and overweight/obesity among school-age children are scarce, so the present study was done to shade a light on the subject.

**Methods:**

This study was a case-control study, conducted on school-age (7-13 years) children. Cases were healthy children who had a body mass index (BMI) percentile of≥85^th^ for age and sex (n=102). Sex-matched children with a BMI percentile between 5^th^ and 85^th^ were considered as control group (n=102). Dietary data were collected using a validated 168-item food frequency questionnaire. Sleep quality was assessed by Pittsburgh sleep quality index. Binary logistic regression was used to assess the association between DPs, sleep quality, and overweight/obesity.

**Results:**

Three DPs were identified: "Low-energy healthy", "High-energy healthy" and "Unhealthy diet". Adherence to the first and second DPs was associated with 51%-62% lower odds of overweight/obesity (Odds ratio [OR]: 0.49, 95% CI: 0.24-0.97, and 0.38, 95% CI: 0.15-0.94, respectively, *P*<0.050). However, we found no significant association for the third DP with overweight/obesity. Furthermore, there was no significant association between sleep quality/duration and overweight/obesity. The interactions of DPs and sleep quality/duration with overweight/obesity were not significant.

**Conclusion:**

Eating a diet high in white meats, eggs, vegetables, fruits and juices, nuts, dairy products, whole grains, and low in refined grains and snacks is associated with a lower likelihood of overweight/obesity in children. This inverse association does not depend on sleep quality/duration.

## Introduction

 The global age-standardized prevalence of obesity is estimated up to 5.6% among girls and 7.8% among boys.^1^ The results of the fifth school-based surveillance program in 2015 showed that in our country, at least 20.6% of children and adolescents were obese and overweight.^2^ Obesity in children is associated with an increased risk of chronic diseases and mortality in adulthood.^3^ In addition, childhood obesity imposes a high economic burden on the health care system. According to a systematic review and meta-analysis by Ling et al, the total annual medical costs of childhood overweight and obesity worldwide have increased to $237.55 per capita in 2022.^4^ Overweight and obesity cause a per capita increase of $70.79, $46.38, and $1975.06 for costs in nonhospital settings, medication, and hospitalization, respectively. Overweight and obesity increase up to 0.28 days length of hospitalization.^4^ Therefore, finding the contributing factors to the incidence of obesity among children is necessary. A combination of genetic and environmental factors is involved in the etiology of childhood obesity.^5^ Among environmental factors, diet plays an important role. Previous studies have shown that intakes of fast foods, calorie-dense snacks, French fries, sweetened beverages, sweets and desserts are positively associated with overweight/obesity in children,^6^ while intakes of high-fiber foods such as vegetables and legumes have a protective role against overweight and obesity.^7^ It should be noted that previous studies have mainly focused on individual food groups and little attention has been paid to dietary patterns (DPs). Interactions between foods and nutrients may influence the association of food and nutrients with childhood obesity. In the DP approach to assess the relationship between diet and disease, these interactions are considered.^8-11^ For example, in a cross-sectional study on 8252 Mexican children aged 5-11 years, following a “Western diet”, containing a high amount of soft drinks, cakes, high-fat foods, tortillas, cereals, sweetened beverages, low-fiber cereals, and salty snacks, was associated with an increased risk of obesity.^12^ In another study on 3843 children aged 8-13 years, Kelishadi et al reported a significant positive association between “sweet DP” (cakes, cookies, pastries, biscuits, chocolate, soft drinks, packaged fruit juice) and obesity.^13^

 In addition to the role of diet, sleep quality/duration may contribute to childhood obesity. It has been shown that insufficient or poor sleep quality might be associated with obesity.^14-16^ In a study on children and adolescents (8-16 years) in China, short sleep duration was positively associated with overweight/obesity, however, no significant association was observed for sleep quality with overweight/obesity.^17^

 Many studies have investigated the relationship between sleep duration and obesity; however, studies on the relationship between sleep quality and overweight/obesity are limited. In addition, most of the studies in this field are from Western countries and there are very limited data on the subject from Asian countries, particularly from -Middle Eastern- countries. There is some evidence on the role of dietary intakes in sleep quality,^18^ but no study investigated the combined association of DPs and sleep quality with overweight/obesity in children. Therefore, this study was conducted to investigate the association of major DPs and sleep quality with odds of overweight/obesity among Iranian school-age children aged 7-13 years.

## Materials and Methods

###  Study design and participants

 This study was a case-control study that was conducted on school children (aged 7-13 years) who were selected from primary schools in Rasht, Iran [April 2021 to December 2022]. Sample size was computed based on the previous research and standard methods of sample size calculation. In pattern determination studies, sample size is usually a multiple of the number of questions or items in the questionnaire. The food frequency questionnaire (FFQ) has a number of food categories based on their nutrients. Usually, the number of food categories in this questionnaire is equal to 40. As a result, the minimum number of samples required is 200 samples (including 5 samples for each category) based on factor analysis to extract patterns.^19,20^ The subjects of the study were selected by a two-stage random sampling method (the first stage was the selection of schools and the second stage was simple random selection of the subjects). In total, 331 primary schools in Rasht are categorized in two areas (1 and 2). Five schools from each area were randomly selected. A Total of ten primary schools (six state schools, and four private schools) were randomly selected. In each school, at least 30 children, from different classrooms were selected and then, those who had inclusion criteria were included in the study. Cases were apparently healthy children who had a body mass index (BMI) percentile of ≥ 85^th^ for age and sex.^21^

 Sex-matched children who had a BMI percentile between 5^th^ and 85^th^were considered as control group. Exclusion criteria were having any types of chronic diseases such as cardiovascular diseases, diabetes, chronic kidney disease, seizure, and genetic diseases, and incomplete data for exposure (dietary intakes and sleep quality) or outcome variables (anthropometric measures).

 Demographic variables including age, gender, birth weight, family size ( ≥ 4 and < 4 persons), first-ranked child (yes/no), family history of obesity (yes/no), and parents’ education were collected through a face-to-face interview. In addition, data on the use of supplements including vitamins D, folic acid, multivitamins, ferrous sulfate, and zinc were collected. In order to assess physical activity, a physical activity questionnaire with 9 levels of activity, from sleep/rest [metabolic equivalents (MET) = 0.9] to vigorous activities (MET > 6) was used. The validity and reliability of this questionnaire has already been confirmed.^22,23^

###  Dietary intake assessment 

 Data on dietary intakes were collected using a semi-quantitative 168-item FFQ that was reliable and valid for Iranian children and adults.^24,25^ FFQ was completed by an experienced nutritionist through a face-to-face interview. The portion sizes of consumed foods were converted to grams using household measures. All subjects daily nutrients intakes were calculated using the US Department of Agriculture’s (USDA) nutrient databank.

###  Assessment of sleep quality/duration

 To assess sleep quality, the Pittsburgh sleep quality index (PSQI) was used.^26^ The PSQI is a self-reported questionnaire. The PSQI indicates how frequently subjects have experienced certain sleep problems over the past month. The total score of this questionnaire, called global sleep score (GSS), can be from 0 to 21. Higher GSS is associated with lower sleep quality. In addition, according to PSQI, good sleep quality is defined as GSS of ˂5 and poor sleep quality as GSS of ≥ 5. Internal consistency and reliability of the PSQI have been tested and verified on a sample of Iranian population in earlier studies (Cronbach’s alpha coefficient = 0.77, sensitivity = 94%, and specificity = 72%).^27,28^In this study, children with sleep ≤ 7 hour/night were defined as short sleeper.^29^

###  Anthropometric measurements

 Children’s weight was measured using a calibrated Seca scale with light clothes, no shoes with an accuracy of 0.1 kg. Children’s height was measured in a standing position without shoes, with an accuracy of 0.1 cm using a wall-mounted tape meter. BMI was calculated from weight and height data.^30^ Normal weight, overweight and obesity were defined according to the BMI percentiles from “Centers for Disease Control and Prevention” (CDC).^21^

###  Statistical analysis

 The study data normality was examined using the Kolmogorov-Smirnov test. Mean and standard deviation were used for continuous variables and frequency and percentage were used for categorical variables.

 To identify DPs, we divided the 168 items of FFQ (except for tea and coffee) into 22 main food groups (Table 1). Then, to determine major DPs, factor analysis with orthogonal transformation (Varimax procedure) was applied. The Kaiser-Meyer-Olkin (KMO) test was used to check whether the distribution of different food groups allowed the use of principal components. Obtained factors were retained for further analysis based on Eigenvalues on the Scree plot. In the study, factors (DPs) with Eigenvalues ≥ 1.8 were retained.

**Table 1:** Food groups used in the factor analysis

**Food groups**	**Food items**
1. Red meat	Lamb, beef, mince-meat, not-processed hamburger
2. Organ meat	Tongue, brain, head, leg, heart, liver, rumen
3. Processed meat	Sausage
4. Fish	Fish, tuna fish
5. Solid oil	Margarine, butter, animal fat, solid vegetable oil
6. Liquid oils	Liquid oil except olive oil
7. Potatoes	Potato, French fries
8. Olives	Green olive, olive oil
9. Fruits and juice	Cantaloupe, melon, watermelon, pear, apricot, cherry, apple, peach, nectar, green tomato, fresh-fig, dried-fig, grape, kiwi, grapefruit, orange, persimmon, tangerine, pomegranate, date palm, plum-red-yellow, sour-cherry, strawberry, banana, lemon, sour-lemon, grapefruit-juice, orange-juice, apple-juice, cantaloupe-juice, cornelian cherry, pineapple-fresh, pineapple-canned, raisins, fresh-mulberry, dried-mulberry, dried-peach, dried-apricot, compote, industrial-juice, lemon-juice and verjuice
10. Low-fat dairy	Low-fat milk, moderate-fat milk, low-fat yogurt, cheese, dough, curd
11. High-fat dairy	High-fat milk, cacao-milk, chocolate milk, strained yogurt, high-fat yogurt, creamy yogurt, creamy cheese, cream, ice cream
12. Nuts	Peanut, almond, walnut, pistachio, hazelnut, seeds
13. Vegetables	Lettuce, tomato, cucumber, mixed vegetables, squash, zucchini, eggplant, celery-cooked, green-peas, green-beans, carrot, garlic, onion, cabbage, spinach, turnip, cooked-mushrooms, green pepper, tomato paste, and red-sauce
14. Snack	Biscuits, crackers, cheese curls, chips
15. Legumes	Lentil, beans, peas, soy beans, mung beans, split peas
16. Whole grains	Whole-grain bread (Sangak), whole-grain toast, barely, bulgur, corn
17. Refined grains	Refined-grain bread (Lavash, Barbari, Tafton, baguette, other breads), rice, pasta, vermicelli, cooked noodles, wheat flour
18. Mayonnaise	Mayonnaise sauce
19. Pickles	Pickle, salty-pickle, pickled-cucumber
20. Chicken and egg	Chicken with skin, skinless-chicken, egg
21. Sugar and dessert	Sugar plum, candy, chocolate, jam, honey, halva (a sweetened breakfast food in Iran), sugar, loaf, creamy-cake, cookie, home-made cake, Yazdi-cake, other cakes, dumpling, home-made halva, cream caramel
22. sugar-sweetened beverages	Industrial juice, carbonated and sweetened drinks

 After the construction of major DPs, children’s DPs scores were classified into tertiles (T). Then, one-way analysis of variance (ANOVA) was used to evaluate the difference in continuous variables including demographic variables and food intakes in tertiles of DPs. The chi-square test was used to assess the distribution of categorical variables across tertiles of DPs’ scores. Crude and adjusted odds ratio (OR) and 95% confidence interval (CI) were calculated using binary logistic regression. For adjusting, three models were used: (I) age and energy intake, (II) age, energy intake, parents’ education, physical activity, and family history of obesity, birth weight, and supplement and (III) age, energy intake, parents’ education, physical activity, and family history of obesity, birth weight, supplement, and sleep quality. To assess the trend of ORs across the increasing tertiles of a DP, dietary patterns were considered as continuous variables. To assess the interactions of DPs’ scores and sleep quality/duration with overweight/obesity, “Generalized Linear Model” (GLM_z_) was used. All statistical analyses were conducted using IBM SPSS Statistics software (version 22) (IBM Corp., Armonk, USA), and *P* values less than 0.05 were considered significant.

## Results

**Figure 1:**
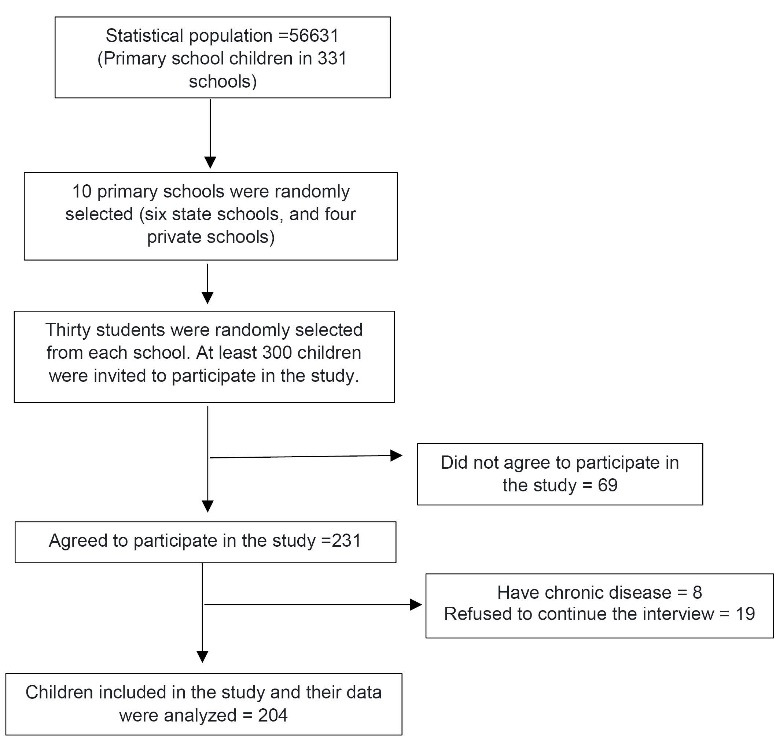
Flow chart of the study

 In total, 204 school children with complete data were included in the study (Figure 1). The studied children had a mean age of 9.56 ± 1.57 years and 50% of them were female. Demographic characteristics, sleep quality, and dietary intakes in children with normal-weight and overweight/obesity are presented in Table 2. Normal-weight children were older and less physically compared to overweight/obese children. The overweight/obese children had higher consumption of whole and refined grains, energy, carbohydrate, fat, calcium, fiber, and polyunsaturated fatty acids (PUFAs) compared to the normal-weight children.

**Table 2:** General characteristics and dietary intakes of overweight/obese and normal weight children

**Variable**s	**Normal-weight (n=102)**	**Overweight/obesity (n=102)**	***P *** **value** ^b^
General characteristics			
Age	9.25 ± 1.50	9.88 ± 1.56	0.003
Gender (female) (%)	50 (49.0)	52 (51.0)	0.770
Birth rank (first) (%)	45 (44.1)	57 (55.9)	0.930
Parents’ education (university educated) (%)	37 (36.3)	44 (43.1)	0.310
PA (Met-h/day)	12.27 ± 4.80	10.26 ± 4.54	0.002
Family history of obesity (yes) (%)	55 (55)	65 (64.4)	0.170
Birth weight (gr)	3215.13 ± 683.81	3191.25 ± 733.15	0.808
Supplement use^a^ (%)	37 (36.6)	30 (29.7)	0.290
Family size ( ≥ 4) (%)	71 (69.6)	66 (64.7)	0.450
Sleep			
Sleep quality (GSS)	1.32 ± 1.75	1.50 ± 1.98	0.502
Sleep timing (o'clock)	10.48 ± 1.99	10.86 ± 1.58	0.133
Sleep duration (h)	8.46 ± 1.15	8.44 ± 1.02	0.897
Sleeping after lunch (yes) (%)	42 (41.2)	45 (44.1)	0.671
Sleep duration after lunch (h)	0.68 ± 0.89	0.73 ± 0.93	0.674
Dietary intakes			
Whole grains (g/d)	42.95 ± 46.26	64.97 ± 67.30	0.007
Refined grains (g/d)	395.40 ± 121.70	446.04 ± 138.86	0.006
Fruits juice (g/d)	206.78 ± 109.87	215.72 ± 164.28	0.640
Vegetables (g/d)	260.81 ± 105.62	264.40 ± 115.88	0.818
Legumes (g/d)	63.72 ± 40.51	64.28 ± 45.32	0.927
Low-fat dairy (g/d)	228.16 ± 154.41	221.91 ± 180.84	0.790
High-fat dairy (g/d)	124.64 ± 108.99	125.55 ± 104.97	0.950
SSB(g/d)	38.63 ± 53.62	37.26 ± 53.72	0.850
Energy (kcal)	2241.12 ± 326.61	2472.05 ± 466.47	≤ 0.001
Protein (g/d)	84.79 ± 19.28	89.50 ± 24.54	0.130
Carbohydrates (g/d)	314.06 ± 54.67	355.84 ± 79.92	≤ 0.001
Fat (g/d)	77.96 ± 13.44	84.37 ± 15.16	0.002
Calcium (mg/d)	1146.29 ± 268.44	1272.25 ± 441.99	0.015
Fiber (g/d)	35.30 ± 8.30	42.7 ± 12.17	≤ 0.001
SFA (g/d)	22.90 ± 5.32	23.88 ± 6.37	0.230
PUFA (g/d)	18.82 ± 3.39	21.16 ± 3.43	≤ 0.001

Data are presented as mean ± standard deviation or percent. Abbreviations: GSS, global sleep score; MET-h, metabolic equivalents-hour; PA, physical activity; PUFA, polyunsaturated fatty acid; SFA, saturated fatty acid; SSB, sugar sweetened beverages. ^a^ Considered as the use of vitamins D, folic acid, multivitamin, ferrous sulfate, zinc. ^b^ Independent sample t test or chi-square test.

 In the factor analysis, three major DPs (DP1, DP2 and DP3) were found.These DPs explained 29.13% of variations in dietary intakes. The value of KMO in this analysis was 0.601, which indicated good sampling adequacy.

 The first DP was characterized by high consumption of chicken, eggs, vegetables, potatoes, low-fat dairy products, pickles, and low consumption of liquid oil (DP1: “Low-energy healthy diet”). The second DP was associated with high consumption of fruits and juices, nuts, high-fat dairy products, fish, olive and olive oil, whole grains and low consumption of refined grains and snacks (DP2: “High-energy healthy diet”). The third DP had high consumption of sugar-sweetened beverages (SSBs), red meat, processed meat and offal, sugar and desserts, mayonnaise, solid oil, and low consumption of legumes (DP3: “Unhealthy diet”).

 The ORs and 95% CIs of overweight/obesity across tertiles of DPs are indicated in Table 3. Adherence to the DP1 was inversely associated with the odds of being overweight/obese. This inverse association remained significant even after adjusting for potential confounders. Children in the highest tertile of DP1 were 80% less likely to be overweight/obese compared to children in the lowest tertile. Such a significant inverse association was also observed for overweight and obesity. Regarding DP2, a significant inverse association was found for overweight/obesity as well as for overweight alone. After controlling for potential confounders, children in the highest tertile of DP2 had 62% and 82% lower odds of being overweight/obese and overweight, respectively, compared with children in the lowest tertile. In terms of adherence to DP3, there was no significant association between DP3 and overweight/obesity.

**Table 3:** Odds ratio and 95% CIs of overweight/obesity across tertiles of dietary patterns in children aged 7-13 years

	**Low-energy healthy diet (DP1)**				**High-energy healthy diet (DP2)**				**Unhealthy diet (DP3)**			
	**T1**	**T2**	**T3**	***P*** *	**T1**	**T2**	**T3**	***P*** *	**T1**	**T2**	**T3**	***P*** *
Overweight/obesity												
Crude	1	0.41 (0.20-0.81)	0.49 (0.24-0.97)	0.040	1	1.07 (0.54-2.08)	0.94 (0.49-1.85)	0.864	1	0.74 (0.38-1.46)	0.79 (0.40-1.55)	0.493
Model 1	1	0.26 (0.12-0.56)	0.21 (0.09-0.50)	< 0.001	1	1.04 (0.51-2.12)	0.76 (0.36-1.61)	0.480	1	0.72 (0.35-1.48)	0.54 (0.26-1.15)	0.110
Model 2	1	0.25 (0.11-0.58)	0.21 (0.08-0.51)	0.001	1	0.74 (0.34-1.60)	0.39 (0.16-0.96)	0.042	1	0.66 (0.31-1.40)	0.53 (0.25-1.15)	0.109
Model 3	1	0.26 (0.11-0.59)	0.20 (0.08-0.51)	0.001	1	0.73 (0.34-1.59)	0.38 (0.15-0.94)	0.037	1	0.66 (0.31-1.40)	0.52 (0.24-1.13)	0.099
Overweight												
Crude	1	0.62 (0.26-1.50)	0.49 (0.20-1.25)	0.139	1	0.77 (0.32-1.88)	0.79 (0.33-1.89)	0.588	1	0.65 (0.26-1.57)	0.72 (0.30-1.73)	0.456
Model 1	1	0.49 (0.19-1.27)	0.35 (0.12-1.00)	0.050	1	0.71 (0.28-1.84)	0.65 (0.24-1.75)	0.387	1	0.59 (0.23-1.540	0.50 (0.19-1.33)	0.166
Model 2	1	0.37 (0.13-1.06)	0.29 (0.09-0.93)	0.035	1	0.38 (0.13-1.12)	0.19 (0.05-0.71)	0.013	1	0.58 (0.21-1.58)	0.48 (0.17-1.35)	0.165
Model 3	1	0.38 (0.13-1.07)	0.29 (0.09-0.93)	0.034	1	0.37 (0.13-1.11)	0.18 (0.05-0.69)	0.012	1	0.58 (0.21-1.59)	0.48 (0.17-1.35)	0.162
Obesity												
Crude	1	0.34 (0.16-0.73)	0.59 (0.29-1.21)	0.136	1	1.42 (0.68-2.97)	1.16 (0.55-2.45)	0.708	1	0.93 (0.45-1.94)	0.93 (0.45-1.94)	0.851
Model 1	1	0.22 (0.10-0.52)	0.27 (0.11-0.65)	0.003	1	1.48 (0.69-3.20)	1.01 (0.44-2.28)	0.977	1	0.95 (0.44-2.06)	0.73 (0.33-1.61)	0.441
Model 2	1	0.26 (0.11-0.74)	0.30 (0.12-0.74)	0.008	1	1.21 (0.53-2.72)	0.78 (0.31-1.98)	0.619	1	1.02 (0.46-2.28)	0.79 (0.35-1.77)	0.563
Model 3	1	0.27 (0.11-0.67)	0.29 (0.12-0.74)	0.007	1	1.21 (0.54-2.74)	0.77 (0.30-1.94)	0.590	1	1.03 (0.46-2.31)	0.78 (0.35-1.76)	0.555

Data are presented as odds ratio (95% CI). Abbreviations: CI, confidence interval; DP, dietary pattern; T, tertile. Model 1: Adjusted for age and energy intake. Model 2: Further adjustments for parents’ education, physical activity, family history of obesity, birth weight, and supplement use. Model 3: Additionally adjusted for sleep quality. ^a^ Binary logistic regression.

 When statistical analysis was performed among children with high sleep quality (GSS < 5), similar findings were found, except for the relationship between DP1 and the odds of obesity. In children with low sleep quality (GSS≥5), there was no significant association between DPs and overweight/obesity. Odds ratios and 95% CIs of being overweight/obese across tertiles of DPs in children with high-quality sleep pattern are presented in Table 4.

**Table 4:** Odds ratio and 95% CIs of overweight/obesity across tertiles of dietary patterns in children with high-quality sleep pattern

	**Low-energy healthy diet (DP1)**				**High-energy healthy diet (DP2)**				**Unhealthy diet (DP3)**			
	**T1**	**T2**	**T3**	***P*** ^a^	**T1**	**T2**	**T3**	***P*** ^a^	**T1**	**T2**	**T3**	***P*** ^a^
Overweight/obesity												
Crude	1	0.44 (0.22-0.91)	0.55 (0.27-1.13)	0.107	1	1.17 (0.58- 2.37)	1.00 (0.49- 2.02)	1.00	1	0.70 (0.35-1.42)	0.94 (0.46-1.90)	0.857
Model 1	1	0.28 (0.12-0.60)	0.24 (0.10-0.57)	0.001	1	1.17 (0.55-2.47)	0.73 (0.33-1.61)	0.457	1	0.64 (0.30-1.38)	0.61 (0.28-1.33)	0.120
Model 2	1	0.28 (0.12-0.66)	0.23 (0.09-0.60)	0.002	1	0.77 (0.34- 1.74)	0.35 (0.13- 0.91)	0.034	1	0.56 (0.25-1.26)	0.54 (0.24-1.21)	0.133
Overweight												
Crude	1	0.56 (0.23-1.39)	0.48 (0.19-1.27)	0.136	1	0.91 (0.36-2.29)	0.86 (0.34-2.14)	0.740	1	0.55 (0.22-1.41)	0.84 (0.34-2.06)	0.685
Model 1	1	0.42 (0.16-1.12)	0.32 (0.11-0.95)	0.039	1	0.89 (0.34-2.38)	0.63 (0.22-1.80)	0.394	1	0.46 (0.17-1.28)	0.55 (0.20-1.51)	0.253
Model 2	1	0.33 (0.11-1.00)	0.24 (0.07-0.84)	0.023	1	0.47 (0.15-1.44)	0.19 (0.05-0.74)	0.017	1	0.45 (0.15-1.32)	0.50 (0.17-1.46)	0.211
Obesity												
Crude	1	0.44 (0.20-0.98)	0.75 (0.35-1.59)	0.431	1	1.44 (0.66-3.11)	1.18 (0.53-2.59)	0.694	1	0.98 (0.45-2.12)	1.08 (0.50-2.33)	0.844
Model 1	1	0.29 (0.12- 0.70)	0.35 (0.14-0.86)	0.022	1	1.51 (0.67-3.41)	0.98 (0.41-2.33)	0.980	1	1.00 (0.44-2.28)	0.79 (0.34-1.81)	0.576
Model 2	1	0.33 (0.13- 0.85)	0.39 (0.15-1.03)	0.052	1	1.16 (0.49-2.79)	0.74 (0.27-2.03)	0.572	1	1.00 (0.42-2.41)	0.78 (0.32-2.41)	0.553

Abbreviations: CI, confidence interval; DP, dietary pattern; T, tertile. Data are presented as odds ratio (95% CI). ^a^ Binary logistic regression. Model 1: Adjusted for age and energy intake. Model 2: Further adjustments for parents’ education, physical activity, family history of obesity, birth weight, and supplement use.

 The ORs and their 95% CIs for the association between sleep quality/duration and overweight/obesity in children are shown in Table 5. The association between sleep quality/duration and odds of being overweight/obese was not significant.

**Table 5:** Odds ratio and 95% CIs for the association between sleep quality/duration and overweight/obesity in children

	**Sleep quality**	**Sleep duration**
	**High** ^a^	**≥7 hours/night** ^a^
Overweight/obesity		
Crude	0.88 (0.32-2.38)	1.63 (0.78-3.42)
Model 1	0.75 (0.27-2.12)	1.41 (0.64-3.11)
Model 2	0.67 (0.23-2.04)	1.06 (0.46-2.43)
Overweight		
Crude	1.08 (0.27-4.28)	1.87 (0.65-5.34)
Model 1	1.11 (0.26-4.69)	2.08 (0.69-6.23)
Model 2	0.85 (0.19-3.71)	1.61 (0.49-5.28)
Obesity		
Crude	0.76 (0.27-2.17)	1.28 (0.56-2.93)
Model 1	0.64 (0.22-1.86)	1.02 (0.43-2.41)
Model 2	0.63 (0.21-1.94)	0.80 (0.32-2.01)

Abbreviations: CI, confidence interval. Data are presented as odds ratio (95% CI). Low sleep quality and short sleep duration (˂ 7 hours/night) were considered as the reference categories for sleep quality and sleep duration, respectively. High and low sleep quality were defined as: global sleep quality score, less than 5, and greater than or equal to 5, respectively. Model 1: Adjusted for age and energy intake. Model 2: Further adjustments for parents’ education, physical activity, family history of obesity, birth weight, and supplement use. ^a^ Binary logistic regression.

 The interactions of DPs’ scores and sleep quality/duration with overweight/obesity among children are presented in Table 6. There was not any interaction between DPs and sleep quality/duration with odds of overweight and obesity.

**Table 6:** Interactions of dietary patterns and sleep quality/duration with overweight/obesity among children aged 7-13 years

	**Interaction between dietary patterns and sleep quality**		**Interaction between dietary patterns and sleep duration**	
	**OR (95% CI)**	***P *** **value** ^a^	**OR (95% CI)**	***P *** **value** ^a^
Overweight/obesity				
DP1*sleep quality/duration	1.04 (0.92-1.17)	0.530	1.09 (0.83-1.44)	0.520
DP2*sleep quality/duration	0.84 (0.67-1.04)	0.110	1.22 (0.86-1.73)	0.270
DP3*sleep quality/duration	0.86 (0.67-1.10)	0.225	1.29 (0.94-1.79)	0.120
Obesity				
DP1*sleep quality/duration	1.05 (0.88-1.25)	0.595	0.83 (0.62-1.10)	0.190
DP2*sleep quality/duration	0.97 (0.84-1.13)	0.738	0.97 (0.68-1.36)	0.840
DP3*sleep quality/duration	1.10 (0.86-1.40)	0.434	0.84 (0.60-1.61)	0.290
Overweight				
DP1*sleep quality/duration	0.86 (0.73-1.01)	0.070	1.97 (0.99-3.93)	0.060
DP2*sleep quality/duration	1.29 (0.98-1.69)	0.070	0.75 (0.48-1.18)	0.210
DP3*sleep quality/duration	1.14 (0.82-1.57)	0.430	0.73 (0.45-1.17)	0.190

Abbreviations: CI, confidence interval; DP, dietary pattern; OR, odds ratio; DP1, Low-energy healthy diet; DP2, High-energy healthy diet; DP3, Unhealthy diet. Data are presented as Odds ratio (95% CI). All ORs were adjusted for age and energy intake, parents’ education, physical activity, family history of obesity, birth weight, and supplement use. ^a^ Generalized linear model.

## Discussion

 There is a correlation between dietary intakes and quality of sleep. There are few data on the association of DPs and sleep quality/duration with overweight/obesity in children. Therefore, in the present study we assessed the association of major DPs and sleep quality/duration with the odds of overweight/obesity in school-age children aged 7-13 years. The study showed that adherence to a diet with ample portions of pickles, chicken, egg, vegetables, potato, low-fat dairy, and a low amount of liquid oil (DP1) is inversely associated with overweight and obesity. The DP1 consisted of a large amount of fiber and calcium, both of which have anti-obesity effects.^7^ Furthermore; high amount of the DP1’s protein content can cause satiety.^31^

 In line with our findings, Oellingrath et al and Zhen et al showed that adherence to the diets rich in vegetables, white meats and meat were associated with reduced odds of overweight/obesity in children and adolescents.^32,33^ However, Kelishadi et al showed that intake of a diet containing large amounts of vegetables, high-fat dairy products, fruits, and fruit juice is not related to obesity in children.^13^ It is possible that obesity-contributing foods, such as: high-fat dairy products and fruit juices are the main causes of these discrepancies.

 In the study, adherence to the DP2 (high intake of fruits and juice, nuts, high-fat dairy, fish, olive and olive oil, whole grains, and low intake of refined grains and snacks) was associated with lower odds of overweight/obesity. In a cross-sectional study of 11- to 17-year-old children, adherence to a DP containing skim dairy products, whole grains, leafy vegetables, fruits, and juices was associated with a lower prevalence of obesity.^34^ In contrast, Yang et al reported that intake of a diet high in whole and refined grains, vegetables, nuts, fruits, dairy products, and egg was not associated with obesity in children aged 11 to 17 years.^35^ As it was mentioned, diets assessed in the previous studies contained different components and this may account for these inconsistent results. In the present study, the DP2 included both obesity-contributing foods (juices, high-fat dairy products, and oils) and anti-obesity foods (whole grains, nuts, and fruits). Despite the presence of obesity-contributing foods in the DP2, the effect of anti-obesity foods seems to be more.

 Previous studies have shown that sleep patterns are related to obesity.^36^ However, in the present study, no significant association between sleep quality/duration, and children’s overweight/obesity was shown. In addition there was not any interaction between DPs and sleep quality/duration with odds of overweight/obesity. These contradicting results may be due to the small number of children with low sleep quality (n = 17) or short sleep (n = 35).

## Strengths and Limitations

 Our study had several strengths. We used regression models that adjusted for potential confounders. In addition, a trained dietician collected dietary data through a validated FFQ. Some limitations should be considered when interpreting our results. Small sample size of students with low sleep quality may be considered as the major limitation for our study. Moreover, although we adjusted for some important confounders, the influence of economic status, region of residence, internet addiction, and psychological disorders was not controlled.

## Conclusion

 In conclusion, DPs rich in protein and fiber are associated with reduced odds of overweight/obesity in children. There is no significant relationship between sleep quality/duration and overweight/obesity. Further studies are needed to assess the interactions of DPs, sleep quality/duration and overweight/obesity in children.

## Acknowledgements

 The authors of the study would like to express their gratitude to the Research Vice-Chancellor of Tabriz University of Medical Sciences, Guilan’s province vice chancellor of Department of Education and also to the children and parents who participated in this study.

## Competing Interests

 Mohammad Asghari Jafarabadi is one of the associate editors of the Health Promotion Perspectives.

## Ethical Approval

 The study was approved by the medical ethics committee of Tabriz University of Medical Sciences, Iran (Registration No: IR. TBZMED. REC.1400.881). Written consent was obtained from the parents of all children.

## References

[1] NCD Risk Factor Collaboration (NCD-RisC) (2017). Worldwide trends in body-mass index, underweight, overweight, and obesity from 1975 to 2016: a pooled analysis of 2416 population-based measurement studies in 128·9 million children, adolescents, and adults. Lancet.

[2] Motlagh ME, Ziaodini H, Qorbani M, Taheri M, Aminaei T, Goodarzi A (2017). Methodology and early findings of the fifth survey of childhood and adolescence surveillance and prevention of adult noncommunicable disease: the CASPIAN-V study. Int J Prev Med.

[3] Gies I, AlSaleem B, Olang B, Karima B, Samy G, Husain K (2017). Early childhood obesity: a survey of knowledge and practices of physicians from the Middle East and North Africa. BMC Pediatr.

[4] Ling J, Chen S, Zahry NR, Kao TA (2023). Economic burden of childhood overweight and obesity: a systematic review and meta-analysis. Obes Rev.

[5] Hernandez-Pacheco N, Kere M, Melén E (2022). Gene-environment interactions in childhood asthma revisited; expanding the interaction concept. Pediatr Allergy Immunol.

[6] Jakobsen DD, Brader L, Bruun JM (2023). Association between food, beverages and overweight/obesity in children and adolescents-a systematic review and meta-analysis of observational studies. Nutrients.

[7] López-Gil JF, Smith L, Abellán-Huerta J, Abellán-Alemán J, Panisello Royo JM, Gutiérrez-Espinoza H (2023). Food consumption patterns related to excess weight and obesity in Spanish preschoolers. Pediatr Res.

[8] Hu FB, Rimm E, Smith-Warner SA, Feskanich D, Stampfer MJ, Ascherio A (1999). Reproducibility and validity of dietary patterns assessed with a food-frequency questionnaire. Am J Clin Nutr.

[9] Jacques PF, Tucker KL (2001). Are dietary patterns useful for understanding the role of diet in chronic disease?. Am J Clin Nutr.

[10] Newby PK, Tucker KL (2004). Empirically derived eating patterns using factor or cluster analysis: a review. Nutr Rev.

[11] Slattery ML (2008). Defining dietary consumption: is the sum greater than its parts?. Am J Clin Nutr.

[12] Rodríguez-Ramírez S, Mundo-Rosas V, García-Guerra A, Shamah-Levy T (2011). Dietary patterns are associated with overweight and obesity in Mexican school-age children. Arch Latinoam Nutr.

[13] Kelishadi R, Heshmat R, Mansourian M, Motlagh ME, Ziaodini H, Taheri M (2018). Association of dietary patterns with continuous metabolic syndrome in children and adolescents; a nationwide propensity score-matched analysis: the CASPIAN-V study. Diabetol Metab Syndr.

[14] Miller MA, Kruisbrink M, Wallace J, Ji C, Cappuccio FP (2018). Sleep duration and incidence of obesity in infants, children, and adolescents: a systematic review and meta-analysis of prospective studies. Sleep.

[15] Taheri S, Lin L, Austin D, Young T, Mignot E (2004). Short sleep duration is associated with reduced leptin, elevated ghrelin, and increased body mass index. PLoS Med.

[16] Miller MA, Bates S, Ji C, Cappuccio FP (2021). Systematic review and meta-analyses of the relationship between short sleep and incidence of obesity and effectiveness of sleep interventions on weight gain in preschool children. Obes Rev.

[17] Chen H, Wang LJ, Xin F, Liang G, Chen Y (2022). Associations between sleep duration, sleep quality, and weight status in Chinese children and adolescents. BMC Public Health.

[18] Ward AL, Reynolds AN, Kuroko S, Fangupo LJ, Galland BC, Taylor RW (2020). Bidirectional associations between sleep and dietary intake in 0-5 year old children: a systematic review with evidence mapping. Sleep Med Rev.

[19] Azadbakht L, Esmaillzadeh A (2008). Dietary and non-dietary determinants of central adiposity among Tehrani women. Public Health Nutr.

[20] Tinsley HE, Brown SD. Handbook of Applied Multivariate Statistics and Mathematical Modeling. San Diego: Academic Press; 2000.

[21] Raymond JL, Morrow K. Krause and Mahan’s Food and the Nutrition Care Process. 16th ed. Elsevier Health Sciences; 2022.

[22] Aadahl M, Jørgensen T (2003). Validation of a new self-report instrument for measuring physical activity. Med Sci Sports Exerc.

[23] Ziaee V, Kelishadi R, Ardalan G, Gheiratmand R, Majdzadeh SR, Motaghian Monazzam M. Physical activity in Iranian students CASPIAN study. Iran J Pediatr 2006;16(2):157-64. [Persian].

[24] Mirmiran P, Hosseini Esfahani F, Mehrabi Y, Hedayati M, Azizi F (2010). Reliability and relative validity of an FFQ for nutrients in the Tehran Lipid and Glucose Study. Public Health Nutr.

[25] Sobhani SR, Pouraram H, Keshtkar A, Dorosti-Motlagh AR. Major dietary patterns and their association with weight status in school age rural children of Bijar, Kordestan. Iran J Nutr Sci Food Technol 2016;11(2):35-46. [Persian].

[26] Buysse DJ, Reynolds CF 3rd, Monk TH, Berman SR, Kupfer DJ (1989). The Pittsburgh Sleep Quality Index: a new instrument for psychiatric practice and research. Psychiatry Res.

[27] Farrahi Moghaddam J, Nakhaee N, Sheibani V, Garrusi B, Amirkafi A (2012). Reliability and validity of the Persian version of the Pittsburgh Sleep Quality Index (PSQI-P). Sleep Breath.

[28] Tavasoli A, Saeidi M, Hooman N (2015). Correlation between sleep quality and blood pressure changes in Iranian children. J Compr Ped.

[29] Grandner MA, Jackson N, Gerstner JR, Knutson KL (2013). Dietary nutrients associated with short and long sleep duration. Data from a nationally representative sample. Appetite.

[30] World Health Organization (WHO). Obesity and Overweight Fact Sheet. WHO; 2016. Available from: https://www.who.int/news-room/fact-sheets/detail/obesity-and-overweight. Accessed June 9, 2021.

[31] Bellissimo N, Fansabedian T, Wong VCH, Totosy de Zepetnek JO, Brett NR, Schwartz A (2020). Effect of increasing the dietary protein content of breakfast on subjective appetite, short-term food intake and diet-induced thermogenesis in children. Nutrients.

[32] Oellingrath IM, Svendsen MV, Brantsaeter AL (2010). Eating patterns and overweight in 9- to 10-year-old children in Telemark county, Norway: a cross-sectional study. Eur J Clin Nutr.

[33] Zhen S, Ma Y, Zhao Z, Yang X, Wen D (2018). Dietary pattern is associated with obesity in Chinese children and adolescents: data from China Health and Nutrition Survey (CHNS). Nutr J.

[34] Pinho L, Silveira MF, Botelho AC, Caldeira AP (2014). Identification of dietary patterns of adolescents attending public schools. J Pediatr (Rio J).

[35] Yang Y, Hu XM, Chen TJ, Bai MJ (2016). Rural-urban differences of dietary patterns, overweight, and bone mineral status in Chinese students. Nutrients.

[36] Sluggett L, Wagner SL, Harris RL (2019). Sleep duration and obesity in children and adolescents. Can J Diabetes.

